# Champagne

**Published:** 1907-06-29

**Authors:** 


### Editor's Letter-Box.

[Our Correspondents are reminded that prolixity is a great bar to publication, and that brevity of style and conciseness of statement greatly facilitate early insertion.]

### "The Hospital's" Commission on Light Wines.

### CHAMPAGNE.

Our Commission's reply to Mr. Andrè L. Simon's letter, published last week, p. 330, is as follows:—

With regard to Mr. Simon's letter, it is quite correct that in some cases no "liqueur" is added to champagne, but even in these cases it is invariably the custom to add sugar to the cuvé. We cannot agree with Mr. Simon that there is a great deal of champagne in this country in which practically no sugar will be found. Although our report was mainly founded on our own analyses, we must state that in coming to our conclusions we have naturally taken into consideration the whole literature of the subject. The champagnes in which the proportion of sugar is so low as to be comparable to that of a dry red wine are very few. A wine which to a layman may seem to contain "practically no sugar" may, on analysis, present a different appearance to the mind of the investigator.—Ed. The Hospital.

THE HOSPITAL *June* 29, 1907.

